# Supplementary material for: Left ventricular reverse remodeling: A predictor of survival in chagasic cardiomyopathy patients with a reduced ejection fraction
Source: PLoS Negl Trop Dis. 2025 Apr 23;19(4):e0013053. doi: 10.1371/journal.pntd.0013053 (PMC12064014; doi:10.1371/journal.pntd.0013053)
Supplement: S1 Table — (PDF) [file pntd.0013053.s001.pdf]

Table S1–Clinical characteristics and comorbidities of the 1043 patients analyzed for the occurrence of reverse remodeling of the left ventricle–T1 (baseline)

Variable	Total (n)*	All patients	PRR (n)*	PRR	NRR (n)*	NRR	P value
Age (years)	1043	57 (48–64)	221	59 (53–66)	822	56 (47–64)	<0.001
Sex [M (%)]	1043	589 (56.5)	221	104 (48.4)	822	482 (58.6)	0.007
BMI (kg/m^2)	832	24.2 (22.2–27.3)	174	24.5 (22.6–27.7)	658	24.2 (22.2–26.0)	0.083
Functional Class (NYHA) [n (%)]	545		111		434		0.650
I		184 (33.8)		37 (33.3)		147 (33.9)	
II		251 (46.1)		48 (43.2)		203 (46.8)	
III		94 (17.2)		21 (18.9)		73 (16.8)	
IV		16 (2.9)		5 (4.5)		11 (2.5)	
Time of symptoms (months)	285	12 (4–36)	55	9 (3–36)	230	12 (4–36)	0.291
HR (bpm)	546	68 (60–76)	116	70 (60–80)	430	65 (60–75)	0.002
SBP (mmHg)	550	110 (100–120)	118	120 (110–130)	432	110 (100–120)	<0.001
DBP (mmHg)	550	70 (60–80)	118	80 (69–82)	432	70 (60–80)	<0.001
**Comorbidities [n (%)]**							
SAH	1043	390 (37.4)	221	96 (43.4)	822	294 (35.8)	0.036
Diabetes Mellitus	1043	170 (16.3)	221	41 (18.6)	822	129 (15.7)	0.307
Hypothyroidism	1043	196 (18.8)	221	39 (17.6)	822	157 (19.1)	0.624
Dyslipidemia	1043	212 (20.3)	221	49 (22.2)	822	163 (19.8)	0.442
COPD/Asthma	1043	26 (2.5)	221	9 (4.1)	822	17 (2.1)	0.090
Smoking	1043	221 (21.2)	221	46 (20.8)	822	175 (21.3)	0.878

Alcoholism	1043	90 (8.6)	221	14 (6.3)	822	76 (9.2)	0.171
Stroke/TIA	1033	222 (21.5)	220	48 (21.8)	813	174 (21.4)	0.894
AMI	1043	38 (3.6)	221	3 (1.4)	822	35 (4.3)	0.041
Atrial Arrhythmias (AF, Atrial Flutter, AT)	1043	355 (34.0)	221	91 (41.2)	822	264 (32.1)	0.012

Data are presented as number of patients and percentages or median values with interquartile ranges (p25–p75)

*n: number of patients with available data for the variable analyzed in the total sample and by groups

PRR: positive reverse remodeling; NRR: negative reverse remodeling; M: male; BMI: body mass index; NYHA: New York Heart Association; SBP: systolic blood pressure; DBP: diastolic blood pressure; HR: heart rate; SAH: systemic arterial hypertension; COPD: chronic obstructive pulmonary disease; CVA: cerebrovascular accident, AMI: acute myocardial infarction; AF: atrial fibrillation; AT: atrial tachycardia.
