# Supplementary material for: Left ventricular reverse remodeling: A predictor of survival in chagasic cardiomyopathy patients with a reduced ejection fraction
Source: PLoS Negl Trop Dis. 2025 Apr 23;19(4):e0013053. doi: 10.1371/journal.pntd.0013053 (PMC12064014; doi:10.1371/journal.pntd.0013053)
Supplement: S2 Table — (PDF) [file pntd.0013053.s002.pdf]

Table S2–Laboratory tests, electrocardiogram and Holter monitoring of the 1043 patients analyzed for the occurrence of reverse remodeling of the left ventricle–T1 (baseline)

Variable	Total (n)*	All patients	PRR (n)*	PRR	NRR (n)*	NRR	P value
**Laboratory Exams**							
Hemoglobin (g/dL)	944	14.2 (13.0–15.2)	195	14.2 (13.1–15.2)	749	14.2 (13.0–15.2)	0.572
Sodium (mEq/L)	915	140 (138–141)	188	140 (138–142)	727	140 (138–141)	0.122
Potassium (mEq/L)	919	4.5 (4.2–4.8)	188	4.5 (4.2–4.8)	731	4.5 (4.2–4.8)	0.438
eGFR (ml/min)†	923	71.1 (56.4–86.7)	186	72.0 (57.1–86.2)	737	71.0 (56.3–86.8)	0.859
Creatinine (mg/dL)	923	1.1 (0.9–1.3)	186	1.1 (0.9–1.3)	737	1.1 (0.9–1.3)	0.041
Urea (mg/dL)	745	41 (33–51)	147	38 (31–48)	598	42 (33–52)	0.061
BNP (pg/mL)	221	472 (177–968)	46	489 (170–1136)	175	472 (181–951)	0.586
**ECG**	631		133		498		
RBBB		249 (39.5)		47 (35.3)		202 (40.6)	0.274
LBBB		69 (10.9)		13 (9.8)		56 (11.2)	0.629
LAFB		217 (34.4)		35 (26.3)		182 (36.5)	0.027
PVC		190 (30.1)		37 (27.8)		153 (30.7)	0.517
SPB		40 (6.3)		8 (6.0)		32 (6.4)	0.863
AVBs		133 (21.1)		15 (11.3)		118 (23.7)	0.002
1st degree AVB		123 (19.5)		14 (10.5)		109 (21.9)	0.003
2nd degree AVB		1 (0.2)		0 (0.0)		1 (0.2)	0.491
3rd degree AVB		10 (1.6)		1 (0.8)		0 (1.8)	0.347
Low QRS voltage		39 (6.2)		7 (5.3)		32 (6.4)	0.621
**24-hour Holter**	588		449		139		
Number of PVC in 24 hours		2458 (655–5976)		1525 (465–5073)		2870 (741–6547)	0.002

PVC in 24 hours (%)	2.8 (0.8–6.6)	1.6 (0.5–5.1)	3.2 (0.9–7.2)	0.002

Data are presented as number of patients and percentages or median values with interquartile ranges (p25–p75)

*n: number of patients with available data for the variable analyzed in the total sample and by groups

†Calculated by CKD-EPI (ml/min)

PRR: positive reverse remodeling; NRR: negative reverse remodeling; eGFR: estimated glomerular filtration rate; BNP: brain natriuretic peptide; ECG: electrocardiogram; RBBB: right bundle branch block; LBBB: left bundle branch block; LAFB: *left anterior fascicular block*; PVC: premature ventricular contraction; SPB: supraventricular premature beat; EESV: supraventricular extrasystoles; ABV: atrioventricular block.
