# Supplementary material for: Left ventricular reverse remodeling: A predictor of survival in chagasic cardiomyopathy patients with a reduced ejection fraction
Source: PLoS Negl Trop Dis. 2025 Apr 23;19(4):e0013053. doi: 10.1371/journal.pntd.0013053 (PMC12064014; doi:10.1371/journal.pntd.0013053)
Supplement: S3 Table — (PDF) [file pntd.0013053.s003.pdf]

Table S3–Treatment of the 1043 patients analyzed for the occurrence of reverse remodeling of the left ventricle–T1 (baseline).

Variable	Total (n)*	All patients	PRR (n)*	PRR	NRR (n)*	NRR	P value
**Treatment**							
ACEi/BRA/ARNI use [n (%)]	577	512 (88.7)	123	107 (87.0)	454	405 (89.2)	0.491
ACEi/ARB dose (mg/day)							
Enalapril	334	20.0 (10.0–40.0)	63	20.0 (10.0–40.0)	271	20.0 (10.0–40.0)	0.329
Captopril	44	75.0 (40.6–75.0)	7	75.0 (50.0–150.0)	37	50.0 (37.5–75.0)	0.530
Losartan	126	100.0 (75.0–100.0)	34	87.5 (50.0–100.0)	92	100.0 (50.0–100.0)	0.175
BB use [n (%)]	578	490 (84.8)	124	95 (76.6)	454	395 (87.0)	0.004
BB dose (mg/day)							
Carvedilol	457	25.0 (12.5–50.0)	81	25.0 (12.5–50.0)	376	25.0 (12.5–50.0)	0.698
Metoprolol Tartrate	11	100.0 (50.0–200.0)	5	200.0 (125.0–200.0)	6	100.0 (50.0–125.0)	0.118
Metoprolol Succinate	4	75.0 (31.3–175.0)	1	25.0 (25.0–25.0)	3	100.0 (50.0 - *)	0.180
Atenolol	13	50.0 (25.0–75.0)	6	50.0 (25.0–100.0)	7	50.0 (25.0–50.0)	0.759
Spironolactone use [n (%)]	576	317 (55.0)	122	47 (38.5)	454	270 (59.5)	<0.001

Spironolactone dose (mg/day)		25.0 (25.0–25.0)		25.0 (25.0–25.0)		25.0 (25.0–25.0)	0.107
Furosemide use [n (%)]	575	351 (61.0)	122	60 (49.2)	453	291 (64.2)	0.002
Furosemide dose (mg/day)		40.0 (40.0–60.0)		40.0 (40.0–40.0)		40.0 (40.0–60.0)	0.889
Thiazide use [n (%)]	576	94 (19.3)	122	21 (17.2)	454	73 (16.1)	0.764
Thiazide dose (mg/day)		25.0 (25.0–25.0)		25.0 (25.0–25.0)		25.0 (25.0–25.0)	0.100
Hydralazine use [n (%)]	576	38 (6.6)	122	5 (4.1)	454	33 (7.3)	0.210
Hydralazine dose (mg/day)		87.5 (75.0–150.0)		112.5 (62.5–300.0)		87.5 (75.0–150.0)	0.619
Nitrate use [n (%)]	576	37 (6.4)	122	4 (3.3)	454	33 (7.3)	0.111
Nitrate dose (mg/day)		60.0 (40.0–80.0)		70.0 (20.0–120.0)		60 (40.0–80.0)	0.801
Digoxin use [n (%)]	576	85 (14.8)	122	15 (12.3)	454	70 (15.4)	0.388
Digoxin dose (mg/day)		0.1 (0.1–0.3)		0.1 (0.1–0.3)		0.3 (0.1–0.3)	0.048
Amiodarone use [n (%)]	576	131 (22.7)	122	17 (13.9)	454	114 (25.1)	0.009
Amiodarone dose (mg/day)		200.0 (200.0–200.0)		200.0 (200.0–200.0)		200.0 (200.0–200.0)	0.993
Triple Therapy use [n (%)] †	577	267 (46.3)	123	38 (30.9)	454	229 (50.4)	<0.001

Data are presented as number of patients and percentages or median values with interquartile ranges (p25–p75)

*N: number of patients with available data for the variables analyzed in the total sample and by groups

†Triple therapy: ACEi/ARB/NIRA, BB and Spironolactone

PRR: positive reverse remodeling; NRR: negative reverse remodeling; ACEi: angiotensin-converting enzyme inhibitors; ARB: angiotensin receptor blockers; ARNI: angiotensin receptor/neprilysin inhibitor; BB: beta blocker.
