# Supplementary material for: Left ventricular reverse remodeling: A predictor of survival in chagasic cardiomyopathy patients with a reduced ejection fraction
Source: PLoS Negl Trop Dis. 2025 Apr 23;19(4):e0013053. doi: 10.1371/journal.pntd.0013053 (PMC12064014; doi:10.1371/journal.pntd.0013053)
Supplement: S4 Table — (PDF) [file pntd.0013053.s004.pdf]

Table S4–Comparison between the first transthoracic echocardiogram of the 1043 patients analyzed for the occurrence of reverse remodeling of the left ventricle–T1 (baseline)

Variable	Total (n)*	All patients	PRR (n)*	PRR	NRR (n)*	NRR	P value
**First TTE**							
LVEF (%)	1043	30.0 (25.0–35.0)	221	30.0 (26.0–35.0)	822	29.0 (25.0–34.0)	<0.001
LVEDD (mm)	1041	63 (59–68)	220	60 (55–64)	821	64 (60–70)	<0.001
LVESD (mm)	1023	54 (49–60)	216	50 (45–54)	807	55 (50–61)	<0.001
LAD (mm)	1030	44 (40–48)	218	43 (39–48)	812	44.5 (41–49)	0.004
RV dysfunction [n (%)]	1035		219		816		0.553
Absent		515 (49.8)		106 (48.4)		409 (50.1)	
Light		224 (21.6)		54 (24.7)		170 (20.8)	
Moderate		188 (18.2)		40 (18.3)		148 (18.1)	
Severe		108 (10.4)		19 (8.7)		89 (10.9)	
Moderate or severe MR [n (%)]	996	478 (48.0)	209	79 (37.8)	787	399 (50.7)	<0.001
Moderate or severe TR [n (%)]	873	245 (28.1)	187	56 (29.9)	686	189 (27.6)	0.518
PASP (mmHg)	607	38 (31–48)	130	35.5 (31–43.3)	477	40 (30.5–48.5)	0.022

Data are presented as number of patients and percentages or median values with interquartile ranges (25–75)

*N: number of patients with available data for the variables analyzed in the total sample and by groups

PRR: positive reverse remodeling; NRR: negative reverse remodeling; TTE: transthoracic echocardiogram; LVEF: left ventricular ejection fraction; LVEDD: left ventricular end-diastolic diameter; LVESD: left ventricular end-systolic diameter; LAD: left atrium diameter; RV: right ventricle; MR: mitral regurgitation; TR: tricuspid regurgitation; PASP: pulmonary artery systolic pressure.
