# Supplementary material for: Left ventricular reverse remodeling: A predictor of survival in chagasic cardiomyopathy patients with a reduced ejection fraction
Source: PLoS Negl Trop Dis. 2025 Apr 23;19(4):e0013053. doi: 10.1371/journal.pntd.0013053 (PMC12064014; doi:10.1371/journal.pntd.0013053)
Supplement: S6 Table — (PDF) [file pntd.0013053.s006.pdf]

Table S6–Treatment of 1043 patients at the time of group allocation to analyze the occurrence of left ventricular reverse remodeling–T2 (follow-up)

Variable	Total (n)*	All patients	PRR (n)*	PRR	NRR (n)*	NRR	P value
**Treatment:**							
ACEi/BRA/ARNI use [n (%)]	1039	903 (86.9)	221	194 (87.8)	818	709 (86.7)	0.665
ACEi/BRA/ARNI dose (mg/day)							
Enalapril	592	20.0 (10.0–40.0)	124	20.0 (10.0–40.0)	468	20.0 (10.0–40.0)	0.171
Captopril	35	75.0 (50.0–150.0)	6	112.5 (65.6–150.0)	29	75.0 (50.0–150.0)	0.287
Losartan	269	100.0 (50.0–100.0)	63	100.0 (50.0–100.0)	206	75.0 (50.0–100.0)	0.455
Sacubitril-Valsartan	2	100.0 (100.0–100.0)	-	-	2	100.0 (100.0–100.0)	-
BB use [n (%)]	1041	995 (95.6)	221	210 (95.0)	820	785 (95.7)	0.649
BB dose (mg/day)							
Carvedilol	968	50.0 (25.0–50.0)	202	50.0 (25.0–50.0)	766	50.0 (25.0–50.0)	0.671
Metoprolol Tartrate	18	200.0 (87.5–200.0)	7	200.00 (200.0–200.0)	11	200.0 (50.0–200.0)	0.079
Metoprolol Succinate	8	100.0 (50.0–100.0)	2	75.0 (50.0–*)	6	100.0 (50.0–125.0)	0.584
Spironolactone use [n (%)]	1040	691 (66.4)	220	135 (61.4)	820	556 (67.8)	0.072
Spironolactone dose (mg/day)		25.0 (25.0–25.0)		25.0 (25.0–25.0)		25.0 (25.0–25.0)	0.125

Furosemide use [n (%)]	1041	758 (72.8)	221	140 (63.3)	820	618 (75.4)	<0.001
Furosemide dose (mg/day)		40.0 (40.0–80.0)		40.0 (40.0–80.0)		40.0 (40.0–80.0)	0.044
Thiazide use [n (%)]	1041	193 (18.5)	221	47 (21.3)	820	146 (17.8)	0.240
Thiazide dose (mg/day)		25.0 (25.0–25.0)		25.0 (25.0–25.0)		25.0 (25.0–25.0)	0.149
Hydralazine use [n (%)]	1040	210 (20.2)	221	36 (16.3)	819	174 (21.2)	0.103
Hydralazine dose (mg/day)		75.0 (75.0–150.0)		100.0 (75.0–200.0)		75.0 (75.0–150.0)	0.452
Nitrate use [n (%)]	1039	164 (15.8)	220	25 (11.4)	819	139 (17.0)	0.043
Nitrate dose (mg/day)		80.0 (40.0–120.0)		80.0 (50.0–120.0)		60.0 (40.0–120.0)	0.671
Digoxin use [n (%)]	1040	212 (20.4)	220	40 (18.2)	820	172 (21.0)	0.361
Digoxin dose (mg/day)		0.1 (0.1–0.3)		0.1 (0.1–0.1)		0.1 (0.1–0.3)	0.743
Amiodarone use [n (%)]	1040	337 (32.4)	221	60 (27.1)	820	277 (33.8)	0.061
Amiodarone dose (mg/day)		200.0 (200.0–400.0)		200.0 (200.0–400.0)		200.0 (200.0-400.0)	0.622
Triple therapy use [n (%)] †	1040	600 (57.7)	221	118 (53.4)	819	482 (58.9)	0.145
Use of cardiac resynchronizer [n (%)]	1043	162 (15.5)	221	21 (9.5)	822	141 (17.2)	0.005
Use of permanent pacemaker [n (%)]	1043	285 (27.3)	221	73 (33.0)	822	212 (25.8)	0.032
ICD use [n (%)]	1043	204 (19.4)	221	34 (15.4)	822	170 (20.7)	0.078

Data are presented as number of patients and percentages or median values with interquartile ranges (p25–p75)

*N: number of patients with available data for the variables analyzed in the total sample and by groups

†Triple Therapy: ACEi/ARB/NIRA, BB and Spironolactone

PRR: positive reverse remodeling; NRR: negative reverse remodeling; ACEi: angiotensin-converting enzyme inhibitors; ARB: angiotensin receptor blockers; ARNI: angiotensin receptor/neprilysin inhibitor; BB: beta blocker. ICD: implantable cardioverter defibrillator
