# Supplementary material for: Left ventricular reverse remodeling: A predictor of survival in chagasic cardiomyopathy patients with a reduced ejection fraction
Source: PLoS Negl Trop Dis. 2025 Apr 23;19(4):e0013053. doi: 10.1371/journal.pntd.0013053 (PMC12064014; doi:10.1371/journal.pntd.0013053)
Supplement: S7 Table — (PDF) [file pntd.0013053.s007.pdf]

Table S7–Comparison between the second transthoracic echocardiogram of the 1043 patients analyzed for the occurrence of reverse remodeling of the left ventricle–T2 (follow-up)

Variable	Total (n)*	All patients	PRR (n)*	PRR	NRR (n)*	NRR	P value
**Second TTE**							
Time between the 1st and 2nd TTE (years)	1043	2.4 (1.3–3.8)	221	2.3 (1.3–3.8)	822	2.4 (1.3–3.8)	0.606
LVEF (%)	1043	30.0 (25.0–35.0)	221	40.0 (39.5–46.5)	822	27.0 (23.0–31.5)	<0.001
ΔLVEF (%)	1043	0.0 (-3.0–5.0)	221	10.0 (5.0–16.0)	822	0.0 (-4.0–2.0)	<0.001
LVEDD (mm)	1027	64 (59–70)	219	58 (53–62)	808	66 (61–71)	<0.001
ΔLVEDD (mm)	1025	1 (-2–4)	218	-1 (-5–2)	807	1 (-1–4)	<0.001
LVESD (mm)	1014	54.5 (48–61)	218	45 (39.8–50)	796	57 (51–63)	<0.001
ΔLVESD (mm)	994	1 (-3–4)	213	-4 (-9–0)	781	2 (-1–5)	<0.001
LAD (mm)	1026	45 (41–51)	219	42 (38–47)	807	46 (42–51)	<0.001
RV dysfunction [n (%)]	1037		220		817		<0.001
Absent		552 (53.2)		164 (74.5)		388 (47.5)	
Light		252 (24.3)		39 (17.7)		213 (26.1)	
Moderate		148 (14.3)		13 (5.9)		135 (16.5)	
Severe		85 (8.2)		4 (1.8)		81 (9.9)	
Moderate or severe MR [n (%)]	1006	560 (55.7)	205	68 (33.2)	801	492 (61.4)	<0.001
Moderate or severe TR [n (%)]	958	332 (34.7)	192	42 (21.9)	766	290 (37.9)	<0.001
PASP (mmHg)	683	38 (30–48)	89	31 (28–40.8)	551	40 (32–50)	<0.001

Data are presented as number of patients and percentages or median values with interquartile ranges (25–75)

*N: number of patients with available data for the variables analyzed in the total sample and by groups

PRR: positive reverse remodeling; NRR: negative reverse remodeling; TTE: transthoracic echocardiogram; LVEF: left ventricular ejection fraction; LVEDD: left ventricular end-diastolic diameter; LVESD: left ventricular end-systolic diameter; LAD: left atrium diameter; RV: right ventricle; MR: mitral regurgitation; TR: tricuspid regurgitation; PASP: pulmonary artery systolic pressure; ΔLVEF: difference between the LVEF value of the 2nd and 1st TTE; ΔLVEDD: difference between the LVEDD value of the 2nd and 1st TTE; ΔLVESD: difference between the LVESD value of the 2nd and 1st TTE.
