# Supplementary material for: Left ventricular reverse remodeling: A predictor of survival in chagasic cardiomyopathy patients with a reduced ejection fraction
Source: PLoS Negl Trop Dis. 2025 Apr 23;19(4):e0013053. doi: 10.1371/journal.pntd.0013053 (PMC12064014; doi:10.1371/journal.pntd.0013053)
Supplement: S8 Table — (PDF) [file pntd.0013053.s008.pdf]

Table S8–Comparison of medication doses at T1 and T2 in PRR group (N = 89)

	n (%)	T1 dose (mg/day)	n (%)	T2 dose (mg/day)	P
Enalapril	50 (56.2)	20.0 (10.0–40.0)	52 (58.4)	20.0 (10.0–40.0)	0.091
Captopril	6 (6.7)	75.00 (50.0–150.0)	4 (4.5)	112.5 (46.9–150.0)	0.068
Losartan	20 (22.5)	100.0 (50.0–100.0)	24 (27.0)	100.0 (50.0–100.0)	0.160
Carvedilol	61 (68.5)	25.0 (12.5–50.0)	82 (92.1)	50.0 (25.0–50.0)	<0.001
Spironolactone	38 (42.7)	25.0 (25.0–25.0)	56 (62.9)	25.0 (25.0–25.0)	0.040
Furosemide	51 (57.3)	40.0 (40.0–70.0)	57 (64.0)	40.0 (40.0–80.0)	0.197
Thiazide	16 (18.0)	25.0 (25.0–25.0)	21 (23.6)	25.0 (25.0–25.0)	0.233
Hydralazine	4 (4.5)	75.0 (50.0–225.0)	18 (20.2)	150.0 (75.0–262.5)	0.002
Nitrate	3 (3.4)	20.0 (20.0–20.0)	11 (12.4)	120.0 (60.0–120.0)	0.006
Digoxin	12 (13.5)	0.125 (0.125–0.250)	13 (14.8)	0.125 (0.125–0.125)	0.969
Amiodarone	8 (9.0)	200.0 (200.0–200.0)	18 (20.2)	200.0 (200.0–400.0)	0.023

Analysis after *propensity score matching*

Data are presented as number of patients and percentages or median values with interquartile ranges (25–75)

Paired medians were compared using the Wilcox test

PRR: positive reverse remodeling; T1: time 1; T2: time 2
