# Supplementary material for: Left ventricular reverse remodeling: A predictor of survival in chagasic cardiomyopathy patients with a reduced ejection fraction
Source: PLoS Negl Trop Dis. 2025 Apr 23;19(4):e0013053. doi: 10.1371/journal.pntd.0013053 (PMC12064014; doi:10.1371/journal.pntd.0013053)
Supplement: S9 Table — (PDF)\ [file pntd.0013053.s009.pdf]

Table S9–Comparison of medication doses at doses at T1 and T2 in NRR group (N = 89)

	n (%)	T1 dose (mg/day)	n (%)	T2 dose (mg/day)	P
Enalapril	51 (57.3)	20.0 (10.0–40.0)	47 (52.8)	10.0 (10.0–40.0)	0.619
Captopril	7 (7.9)	50.0 (37.5-75.0)	4 (4.5)	112.5 (46.9–150.0)	0.027
Losartan	18 (20.2)	100.0 (50.0–10.0)	21 (23.6)	100.0 (50.0–100.0)	0.829
Carvedilol	68 (76.4)	25.0 (12.5–50.0)	82 (92.1)	50.0 (12.5–50.0)	<0.001
Spironolactone	42 (47.2)	25.0 (25.0–25.0)	59 (66.3)	25.0 (25.0–25.0)	0.004
Furosemide	49 (55.1)	40.0 (40.0–40.0)	61 (68.5)	40.0 (40.0–80.0)	0.001
Thiazide	15 (16.9)	25.0 (25.0–25.0)	11 (12.4)	25.0 (25.0–25.0)	0.691
Hydralazine	8 (9.0)	112.5 (75.0–150.0)	20 (22.5)	75.0 (56.3–206.3)	0.005
Nitrate	6 (6.7)	50.0 (40.0–80.0)	14 (15.7)	60.0 (40.0–120.0)	0.006
Digoxin	9 (10.1)	0.125 (0.125–0.250)	13 (14.6)	0.125 (0.125–0.188)	0.693
Amiodarone	13 (14.6)	200.0 (200.0–350.0)	28 (31.5)	200.0 (200.0–400.0)	<0.001

Analysis after *propensity score matching*

Data are presented as number of patients and percentages or median values with interquartile ranges (25–75)

Paired medians were compared using the Wilcox test

NRR: negative reverse remodeling; T1: time 1; T2: time 2.
