# Supplementary material for: Left ventricular reverse remodeling: A predictor of survival in chagasic cardiomyopathy patients with a reduced ejection fraction
Source: PLoS Negl Trop Dis. 2025 Apr 23;19(4):e0013053. doi: 10.1371/journal.pntd.0013053 (PMC12064014; doi:10.1371/journal.pntd.0013053)
Supplement: S10 Table — (PDF) [file pntd.0013053.s010.pdf]

Table S10–Incidence of primary and secondary outcomes during follow-up

Outcomes	Total (n = 178)	PRR (n = 89)	NRR (n = 89)	P value
Total mortality/heart transplant [n (%)]	65 (36.5)	22 (24.7)	43 (48.3)	0.002
Total mortality [n (%)]	56 (31.5)	19 (21.3)	37 (41.6)	0.004
Heart transplant [n (%)]	9 (5.1)	3 (3.4)	6 (6.7)	0.496

Data are presented as the numbers of patients and percentages

PRR: positive reverse remodeling; NRR: negative reverse remodeling
