# Supplementary material for: Left ventricular reverse remodeling: A predictor of survival in chagasic cardiomyopathy patients with a reduced ejection fraction
Source: PLoS Negl Trop Dis. 2025 Apr 23;19(4):e0013053. doi: 10.1371/journal.pntd.0013053 (PMC12064014; doi:10.1371/journal.pntd.0013053)
Supplement: S11 Table — (PDF) [file pntd.0013053.s011.pdf]

Table S11–Univariate Cox proportional risk analysis of factors with potential impact on event-free survival (total mortality and heart transplant).

	Univariate Analysis		
	**HR**	**95% CI**	**P value**
LVRR Positive*	0.450	0.269–0.753	0.002
NYHA Functional Class (T1) #	1,410	1,075-1851	0.013
Systolic Blood Pressure (every 1 mmHg) (T1)	0.979	0.966–0.992	0.001
Atrial Pressure (every 1 mmHg) (T1)	0.978	0.958–0.998	0.032
Age (every 1 year) (T1)	0.978	0.957–0.999	0.042
Use of Furosemide (T1)	1,870	1.103–3.168	0.020
Use of Hydralazine (T1)	3,696	1,916–7,123	<0.001
Hydralazine Dose (every 1 mg/day) (T1)	1.008	1.003–1.012	<0.001
Furosemide Dose (every 1 mg/day) (T1)	1,009	1.004–1.014	0.001
Nitrate Dose (every 1 mg/day) (T1)	1,011	1,000–1,022	0.042
Age (every 1 year) (T2)	0.973	0.952–0.994	0.011
NYHA Functional Class (T2) #	1,715	1.293–2.275	<0.001
Systolic Blood Pressure (every 1 mmHg) (T2)	0.991	0.984–0.998	0.015
Hemoglobin (every 1 g/dL) (T2)	0.858	0.737–0.999	0.049
Sodium (every 1 mEq/L) (T2)	0.808	0.742–0.881	<0.001
Use of Furosemide (T2)	2,872	1.464–5.636	0.002
Furosemide dose (every 1 mg/day) (T2)	1,011	1.007–1.016	<0.001
Use of Hydralazine (T2)	1.006	1.004–1.009	<0.001
Hydralazine Dose (every 1 mg/day) (T2	1,862	1.099–3.155	0.021
LVEF (every 1%) (T1)	0.921	0.887–0.956	<0.001
LVDD (every 1 mm) (T1)	1,084	1.050–1.119	< 0.001

LVSD (every 1 mm) (T1)	1,078	1.047–1.110	<0.001
LAD (every 1 mm) (T1)	1,047	1.009–1.087	0.016
RV dysfunction (T1) [&]	1,461	1.150–1.855	0.002
Moderate or severe MI (T1)	1,936	11.164–3.222	0.011
Moderate or severe TI (T1)	2,188	1.329–3.602	0.002
LAD (every 1 mm) (T2)	1,048	1.016–1.081	0.003
SPAP (every 1 mmHg) (T2)	1,023	1.003–1.043	0.023
Moderate or severe MI (T2)	2,262	1.372–3.731	0.001
RV dysfunction (T2) [&]	1,792	1.403–2.289	<0.001

The variables were analyzed after *propensity score matching* (n = 178).

[+] Analyzed as categories: positive reverse remodeling, use of furosemide (T1 and T2), use of hydralazine (T1 and T2); use of hydralazine (T1 and T2), moderate or severe Mitral Insufficiency in the 2nd and 1st TTE; Moderate or severe Tricuspid Insufficiency in the 1st TTE

[#] NYHA Functional Class (T1 and T2), analyzed in categories: I, II, III and IV.

[&] Right ventricular (RV) systolic function, classified into the following categories: normal, discreetly harmed, moderately impaired, seriously harmed.

TTE: transthoracic echocardiogram; LVRR: left ventricular e reverse remodeling; NYHA: New York Heart Association; T1: time 1; T2: time 2; LVEF: left ventricular ejection fraction; LVDD: left ventricular end-diastolic diameter; LVSD: left ventricular end-systolic diameter; LAD: left atrium diameter; MI: mitral insufficiency; TI: tricuspid insufficiency; RV: right ventricle; SPAP: systolic pulmonary artery pressure.
