# Supplementary material for: Left ventricular reverse remodeling: A predictor of survival in chagasic cardiomyopathy patients with a reduced ejection fraction
Source: PLoS Negl Trop Dis. 2025 Apr 23;19(4):e0013053. doi: 10.1371/journal.pntd.0013053 (PMC12064014; doi:10.1371/journal.pntd.0013053)
Supplement: S12 Table — (PDF) [file pntd.0013053.s012.pdf]

Table S12– Quartiles of numerical variables with potential impact on event-free survival.

	Q1	Q2	Q3	Q4
LVEDD (mm) in 1st TTE	46–57	58–61	62–66	67–86
Sodium (mEq/L) (T2)	131–137	138–139	140–141	142–145

Q: quartile

LVEDD: left ventricular end-diastolic diameter; TTE: transthoracic echocardiogram; T2: Time 2
