# Supplementary figures and images for: Left ventricular reverse remodeling: A predictor of survival in chagasic cardiomyopathy patients with a reduced ejection fraction
Source: PLoS Negl Trop Dis. 2025 Apr 23;19(4):e0013053. doi: 10.1371/journal.pntd.0013053 (PMC12064014; doi:10.1371/journal.pntd.0013053)

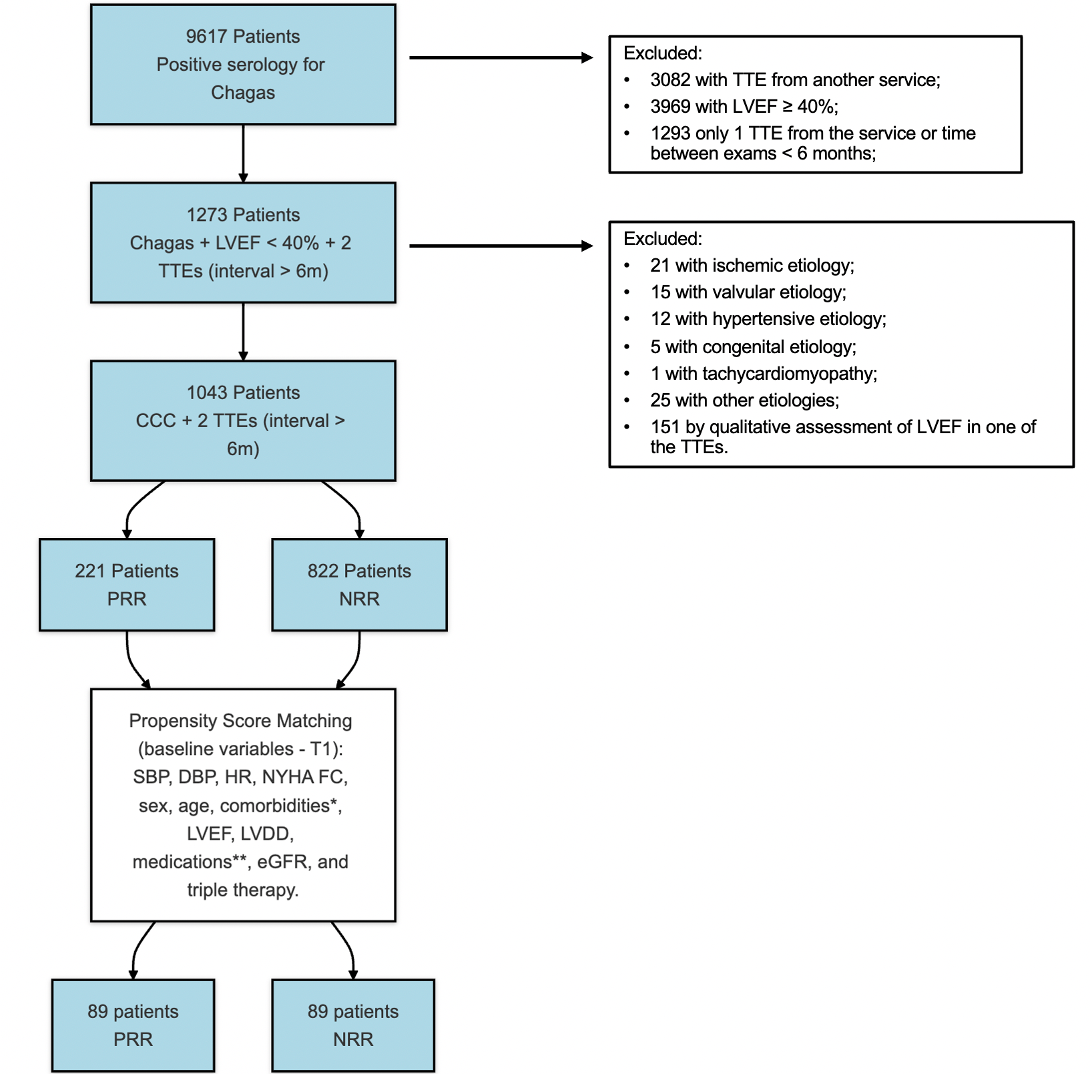

Supplement: S1 Fig — *Systemic arterial hypertension, diabetes mellitus; dyslipidemia; atrial arrhythmias, alcohol use, smoking, chronic obstructive pulmonary disease or asthma, stroke or transient ischemic attack, hypothyroidism, acute myocardial infarction. ** Angiotensin-converting enzyme inhibitors/angiotensin receptor blockers/neprilysin and angiotensin receptor inhibitors, beta-blockers, spironolactone, furosemide, thiazide, hydralazine, nitrate, digoxin, and amiodarone. TTE, transthoracic echocardiography; LVEF, left ventricular ejection fraction; CCC, chronic Chagasic cardiomyopathy; PRR, positive left ventricular reverse remodeling; NRR, negative left ventricular reverse remodeling; SBP, systolic blood pressure; DBP, diastolic blood pressure; HR, heart rate; FC, functional class; NYHA, New York Heart Association; LVDD, left ventricular end-diastolic diameter; eGFR, estimated glomerular filtration rate. (TIF) [file pntd.0013053.s013.tif]

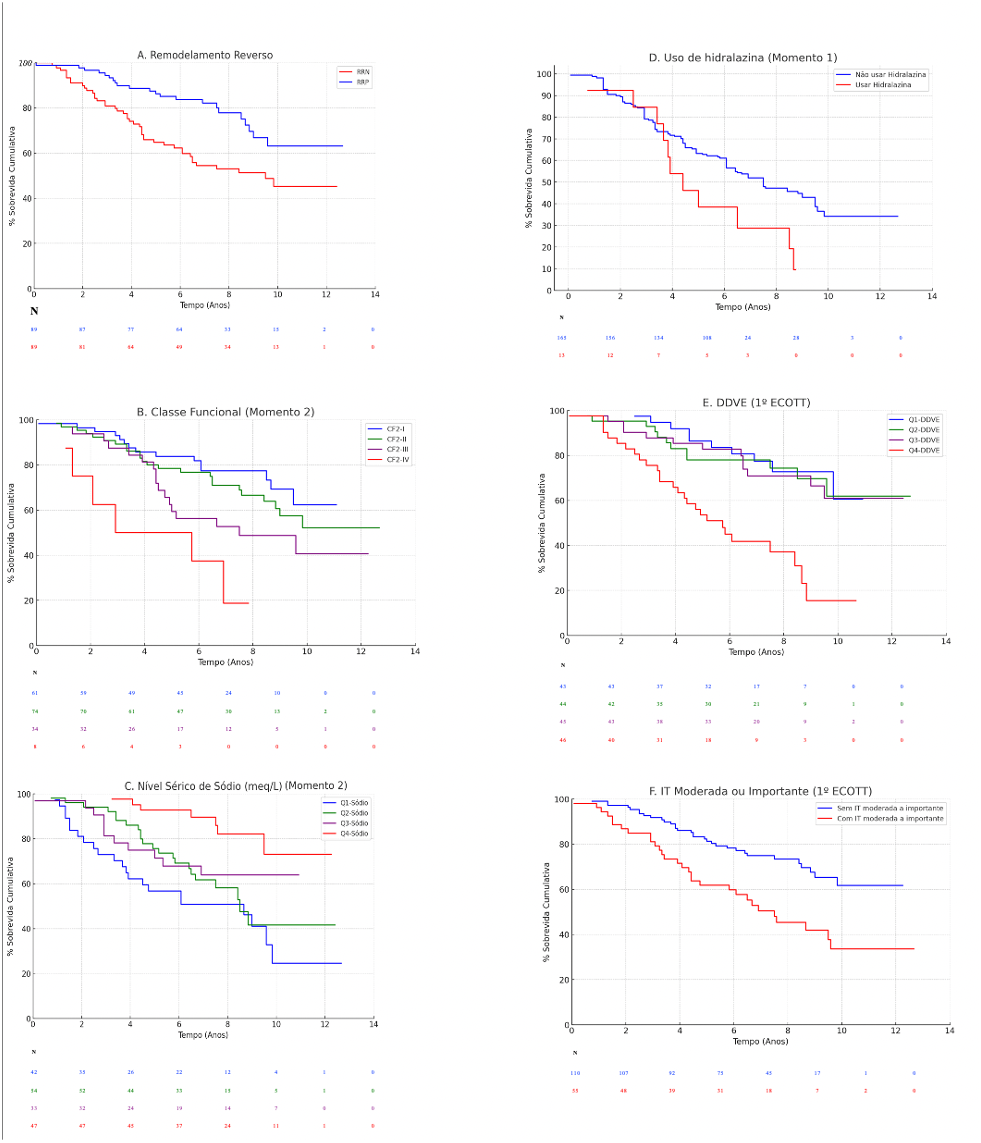

Supplement: S2 Fig — (A) Presence of PRR (B) Functional Class (NYHA) at T2. (C) Serum sodium levels at T2. (D) Use of hydralazine at T1. (E) LVEDD in the first transthoracic echocardiogram (TTE). (F) Presence of moderate or severe tricuspid regurgitation in the first TTE. PRR, positive reverse remodeling; LVRR-, negative left ventricular reverse remodeling; FC2, New York Heart Association Functional Class at moment 2; LVEDD, left ventricular end-diastolic diameter; TR, tricuspid regurgitation; Q1, 1st quartile; Q2, 2nd quartile; Q3, 3rd quartile; Q4, 4th quartile. (TIF) [file pntd.0013053.s014.tif]
