# Supplementary material for: Left ventricular reverse remodeling: A predictor of survival in chagasic cardiomyopathy patients with a reduced ejection fraction
Source: PLoS Negl Trop Dis. 2025 Apr 23;19(4):e0013053. doi: 10.1371/journal.pntd.0013053 (PMC12064014; doi:10.1371/journal.pntd.0013053)
Supplement: Attachment — Submitted filename: Response to Reviewers PLOS .docx [file pntd.0013053.s016.docx]

**Response to Reviewers**

**Manuscript ID:** PNTD-D-25-00077

**Title:** Left ventricular reverse remodeling: a predictor of survival in chagasic cardiomyopathy patients with a reduced ejection fraction

Dear Editor and Reviewers,

We appreciate the opportunity to revise our manuscript and would like to express our gratitude for the constructive feedback provided. Your insightful comments have significantly improved the quality and clarity of our study. Below, we provide detailed responses to each point raised, indicating how we have addressed them in the revised manuscript. All changes are highlighted in the **Revised Manuscript with Track Changes.**

1. **Inclusion of Benznidazole Treatment Data**

- Reviewer’s Comment: The reviewer raised an important point regarding the impact of etiological treatment with Benznidazole (BZN) on left ventricular reverse remodeling (LVRR) and survival in Chagasic cardiomyopathy.
- Response: We acknowledge the reviewer’s valuable observation regarding BZN therapy. However, data on prior BZN administration were not systematically collected in our cohort. Our study aimed to evaluate the association between LVRR and survival in Chagas cardiomyopathy patients undergoing standard heart failure therapy, as currently recommended for patients with heart failure with reduced ejection fraction (HFrEF). In clinical practice, many CCC patients enter specialized heart failure clinics at an advanced disease stage, where BZN treatment may have been administered years earlier, often before referral. Given the retrospective nature of our study, tracking prior BZN use would be challenging and prone to incomplete or unreliable data. Therefore, we opted not to include this variable in our analysis. To address this concern, we have now explicitly mentioned the absence of BZN treatment data and provided our rationale in the Discussion section (page 30, lines 414 - 418).

2. **Rephrasing of CCC Pathogenesis Description**

- Reviewer’s Comment: The reviewer suggested rewording the description of Chronic Chagasic Cardiomyopathy (CCC) pathogenesis to emphasize parasite persistence as a key factor.
- Response: We have revised the sentence accordingly to reflect the role of parasite persistence in CCC pathogenesis, as suggested. The updated text appears in the Introduction (page 6, lines 130 and 131), and we have included Reference 2 to support this statement.

3. **Clarification of Local Guidelines**

- Reviewer’s Comment: The reviewer requested clarification on the “local guidelines” referenced in the Methods section.
- Response: We have now explicitly stated the specific guidelines used for patient management and provided appropriate citations in the Methods section (page 7, line 184).

4. **Inclusion of QT Dispersion Analysis**

- Reviewer’s Comment: The reviewer inquired whether QT dispersion or other ECG parameters were associated with PRR or NRR in our cohort.
- Response: Unfortunately, QT dispersion was not systematically assessed in our study population. However, we acknowledge its prognostic significance in CCC, particularly due to its potential association with chronic inflammation. To address this, we have mentioned this limitation in the Discussion section (page 30, lines 468 - 470) and emphasized the need for future studies to explore this relationship.

5. **Predictors of PRR (Table S13) Should Be Added as a Main Table (Table 4)**

- Reviewer’s Comment: The reviewer suggested incorporating the predictors of PRR into the main manuscript rather than keeping them in the Supplementary Material.
- Response: We agree with this suggestion. The analysis of predictors of PRR identified in our analysis are valuable for the discussion and should be included in the main text. Accordingly, we have moved Table S13 into the main manuscript as Table 4 and referenced it appropriately in the Results section (page 24 and 25, line 338).

6. **Addition of “Potential Clinical Implications” and “Limitations” Sections**

- Reviewer’s Comment: The reviewer recommended adding two new sections: Potential Clinical Implications and Limitations
- Response: We appreciate this valuable suggestion. We have now incorporated these sections at the end of the Discussion (pages 30-31) to clearly outline the real-world implications of PRR and acknowledge the study’s limitations.

7. **Minor Grammar and Formatting Corrections**

- Reviewer’s Comment: The reviewer suggested minor language revisions and noted that "(T. cruzi)" should be omitted from line 104.
- Response: We removed "(T. cruzi)" as recommended.

8. **Clarification on “ETT”**

- Reviewer’s Comment: The reviewer asked if "ETT" in line 139 referred to transthoracic echocardiography (TTE).
- Response: Yes, "ETT" was a typo. We have corrected it to TTE (transthoracic echocardiography).

We sincerely appreciate the reviewers’ thoughtful feedback and suggestions, which have strengthened our manuscript. We believe the revisions have improved the clarity, scientific rigor, and overall quality of our work. Thank you for your time and consideration. We look forward to your further evaluation.

Sincerely,

Maria Tereza Sampaio de Sousa Lira, MDD

PhD Student, Heart Failure Clinics – Heart Institute (INCOR)

University of São Paulo School of Medicine, São Paulo, Brazil
